# Full-length standing radiographs can be used for determination of the Femoral neck-shaft angle but not acetabular coverage

**DOI:** 10.1051/sicotj/2022033

**Published:** 2022-08-24

**Authors:** Sufian S. Ahmad, Christian Konrads, Annika Steinmeier, Max Ettinger, Henning Windhagen, Gregor M. Giebel

**Affiliations:** 1 Department of Orthopaedic Surgery, Hannover Medical School 30625 Hannover Germany; 2 Faculty of Medicine, University of Tübingen 72076 Tübingen Germany; 3 Department of Orthopaedic Surgery, University of Tübingen 72076 Tübingen Germany; 4 Center for Musculoskeletal Surgery, Charité – University Medical Center 10117 Berlin Germany

**Keywords:** Full-length weight-bearing X-ray, Conventional ap pelvic radiograph, Osteotomy, Hip dysplasia, Femoroacetabular impingement, High tibial osteotomy, Distal femur osteotomy

## Abstract

*Introduction*: The exact evaluation of hip morphology is essential for surgical planning. A wide range of morphometric measures of the acetabulum is deduced from conventional anterior-posterior (ap) pelvic radiographs. Full-length weight-bearing radiographs (FLWBR) also depict the acetabulum and are commonly used for osteotomy planning of the lower limb. This study aimed to determine whether FLWBR can be used to evaluate acetabular morphology. *Methods*: Radiographs of patients receiving a hip workup that included a conventional ap pelvic X-ray and FLWBR were utilized for radiographic measurements. The following parameters were measured: extrusion index of the femoral head, anterior wall index, posterior wall index, lateral center edge angle (LCE), acetabular index, pubic arc angle (subpubic angle), and centrum-collum-diaphyseal angle (CCD). *Results*: FLWBR depicted a significantly reduced anterior coverage (*p* = 0.049) and increased posterior coverage (*p* < 0.001), higher acetabular index (*p* = 0.015), and higher pubic-arc angle (*p* = 0.02) compared to conventional ap pelvic radiographs. There were no significant differences regarding the CCD angle (*p* = 0.28), extrusion index (*p* = 0.31), and LCE (*p* = 0.16). *Discussion*: The CCD angle of the femur can be measured on conventional ap radiographs and full-length weight-bearing X-rays for lower limb deformity analysis. However, FLWBR will depict an anteverted acetabular morphology, rendering conventional ap radiographs necessary for planning pelvic osteotomies.

## Introduction

The exact evaluation of hip morphology is essential for surgical planning [1–5]. Many well-established surgical procedures mandate obtaining a conventional anterior-posterior (ap) pelvic X-ray as a pillar of diagnostic radiography [6, 7]. This radiographic image also represents the most validated diagnostic tool [5, 6, 8–11].

A wide range of morphometric measures can be deduced from a conventional ap pelvic radiograph, including the lateral acetabular coverage of the femoral head, anterior and posterior wall coverage of the acetabulum, and extrusion of the femoral head [6]. This information is very valuable in determining hip dysplasia or even focal or global acetabular coverage associated with femoroacetabular impingement [4–6].

Further X-ray modalities are frequently obtained to determine the alignment and morphology of the lower limb. One example is the full-length standing radiograph frequently used for determining the knee alignment, assessing leg length discrepancy, and deformities of a long bone. The radiographs are performed in a standardized fashion and depict the hip, knee, and ankle joints [12].

Given that the hip joint is depicted on full-length weight-bearing radiographs (FLWBR), the authors asked whether these radiographs are sufficient to replace a convention pelvic X-ray. Therefore, this study aimed to determine how radiographic morphometric measures of the hip differ between FLWBR and conventional pelvic X-rays. It was hypothesized that there is a significant difference in the depiction of acetabular orientation between both radiographic modalities.

## Material and methods

### Patients

Radiographs of patients receiving a hip workup that included a conventional ap pelvic X-ray and FLWBR were considered for inclusion in the study, provided consent was received. The radiographic images had to be performed as described below, fulfilling quality criteria. X-rays performed in external institutions and replaced hips were excluded to maintain a standardized setting.

### Radiographs

FLWBR was obtained in accordance with Paley with a 1.3 m cassette (Global Imaging Baltimore, MD) [13], and the patient stood in a bipedal stance in front of the long film cassette. The radiography tube was positioned 305 cm away, and the selected film cassette was of sufficient length to include the hips, knees, and ankles. The magnification with this setup was approximately 5%. A magnification device (250 mm steel ball) was used to calibrate the radiographs. The X-ray beam was centered at the level of the knee joints.

Radiologic technical assistants were instructed to position both legs with the patella centered between the femoral condyles. It was of utmost importance to ensure standardized radiography.

Conventional ap pelvic X-rays were performed in a standardized manner by placing the film focus at a distance of 1.2 m from the pelvis. The central beam was directed at the intersection point of two lines, one connecting both anterosuperior iliac spines and a line intersecting the superior border of the symphysis.

### Radiographic parameters

**Figure 1:**
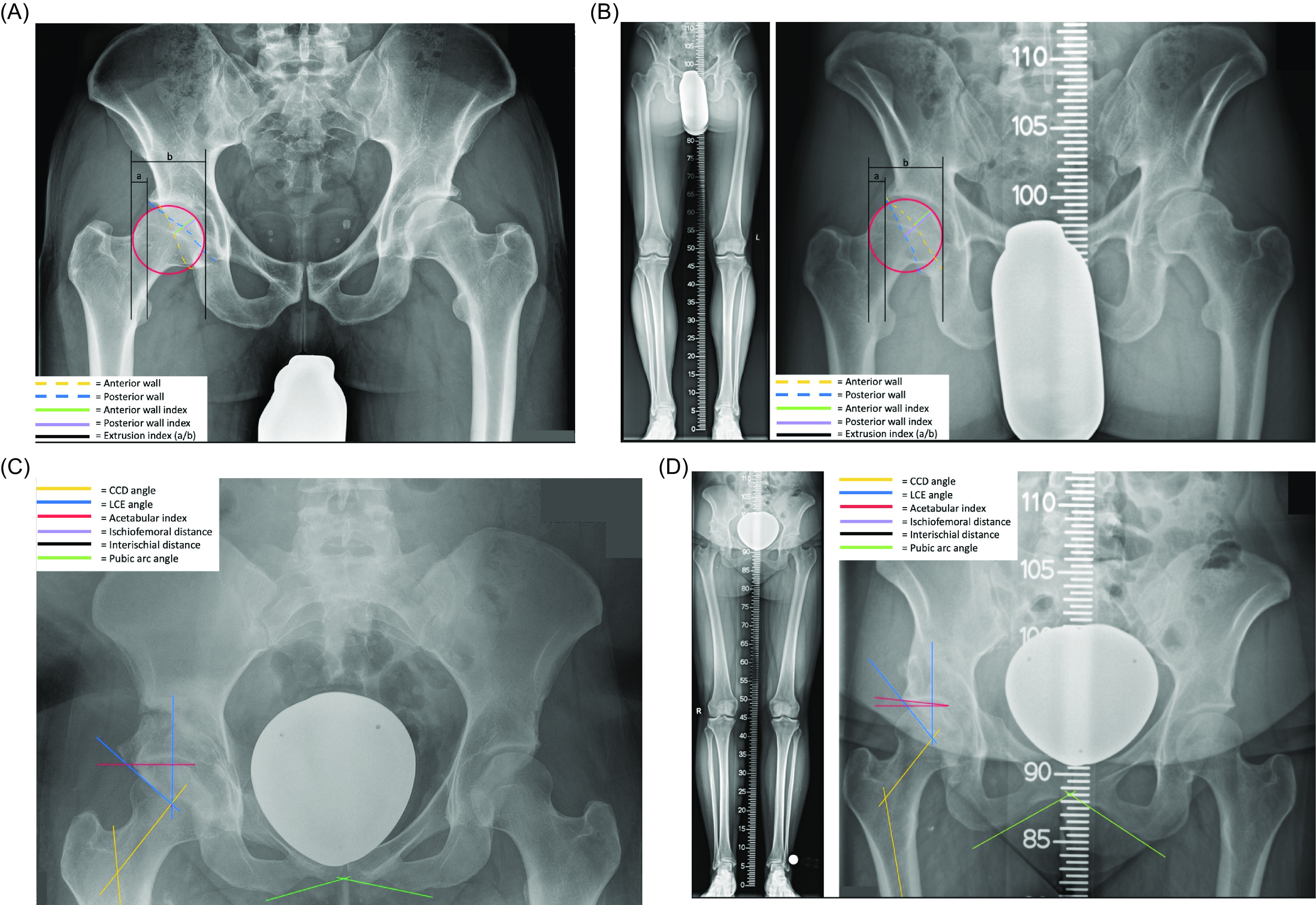
(a–d): Illustration of radiographic measures used for morphometric analysis of the hip in conventional ap pelvic X-rays and full-length weight-bearing radiographs. CCD: centrum collum diaphyseal angle, LCE: lateral center edge angle.

Radiographic parameters were determined with an accuracy of 0.1 mm using mediCAD^®^ (Hectec, Landshut, Germany). The following parameters were assessed (Figure 1):

- Extrusion index of the femoral head
- Anterior wall index
- Posterior wall index
- Lateral center edge angle (LCE)
- Acetabular index
- Pubic arch angle (subpubic angle)
- Centrum-Collum-diaphyseal angle (CCD)

### Statistical analysis

Continuous variables were presented as median (interquartile range). Comparison between medians was performed using the Wilcoxon–Mann–Whitney-Test. A *P*-value of < 0.05 was considered statistically significant. SPSS version 24 (IBM, Armonk, New York) was utilized for analysis.

Ethical approval was received for the conduction of this study 421/2020BO.

## Results

Considering the above criteria, the radiographs of 52 limbs of 52 individual patients (30 females and 22 males) with a mean age of 36.5 ± 15.0 years were included in this analysis.

The CCD angle did not significantly differ between FLWBR and ap pelvic X-rays. The mean CCD angle measures on ap pelvic radiographs were 135.4° ± 8.7°, and on FLWBR, it was 135.0° ± 8.6° (*p* = 0.28).

The LCE angle did not significantly differ between both radiographic methods. FLWBR depicted a slightly lower median LCE of 34.7° (Interquartile range (IQR) 29.3° – 39.9°) compared to the conventional ap pelvic X-ray with a median LCE of 38.0° (IQR 30.9° – 44.0°) (*p* = 0.16).

**Figure 2:**
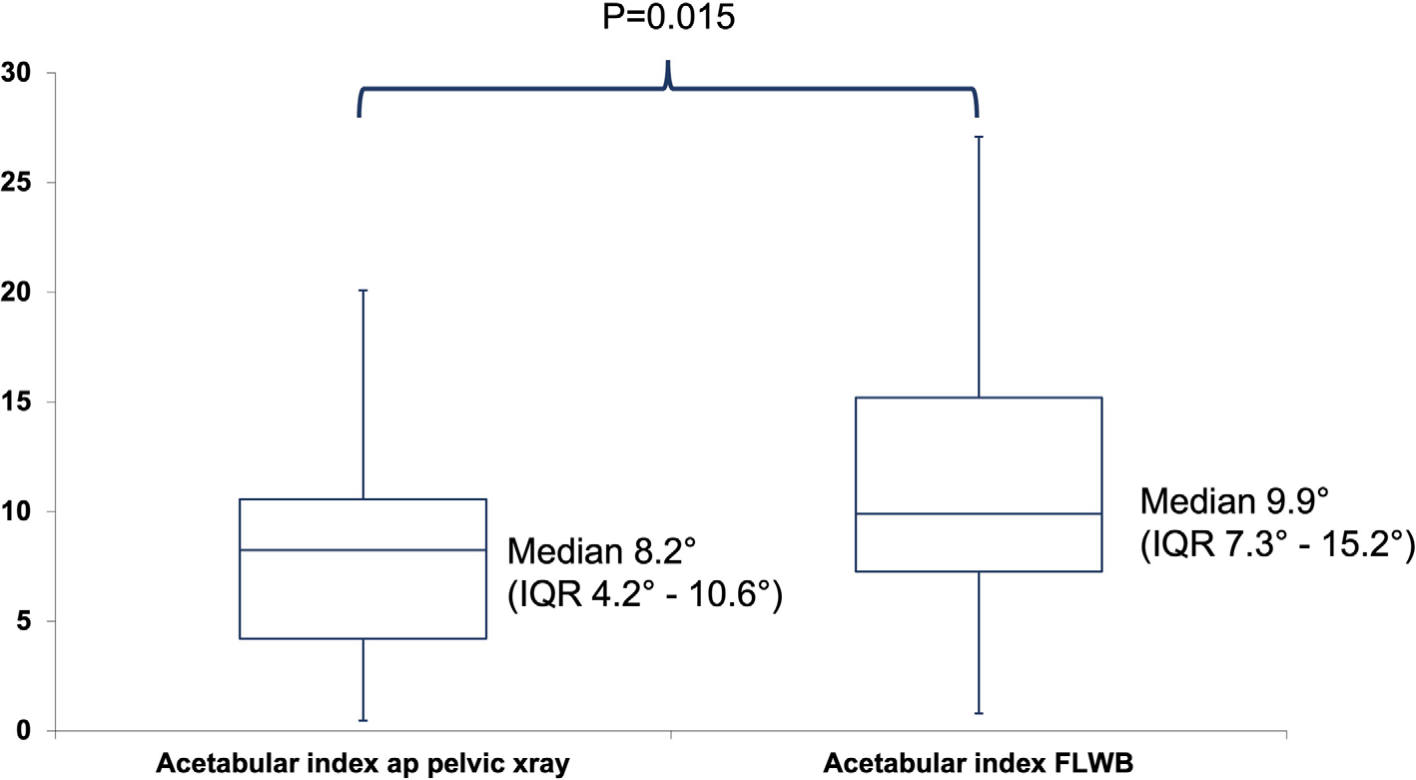
Box plot depicting the acetabular index measured on conventional ap pelvic radiographs and full-length weight-bearing radiographs.

The acetabular index was significantly higher in FLWBR compared to conventional ap pelvic X-ray (Figure 2).

**Figure 3:**
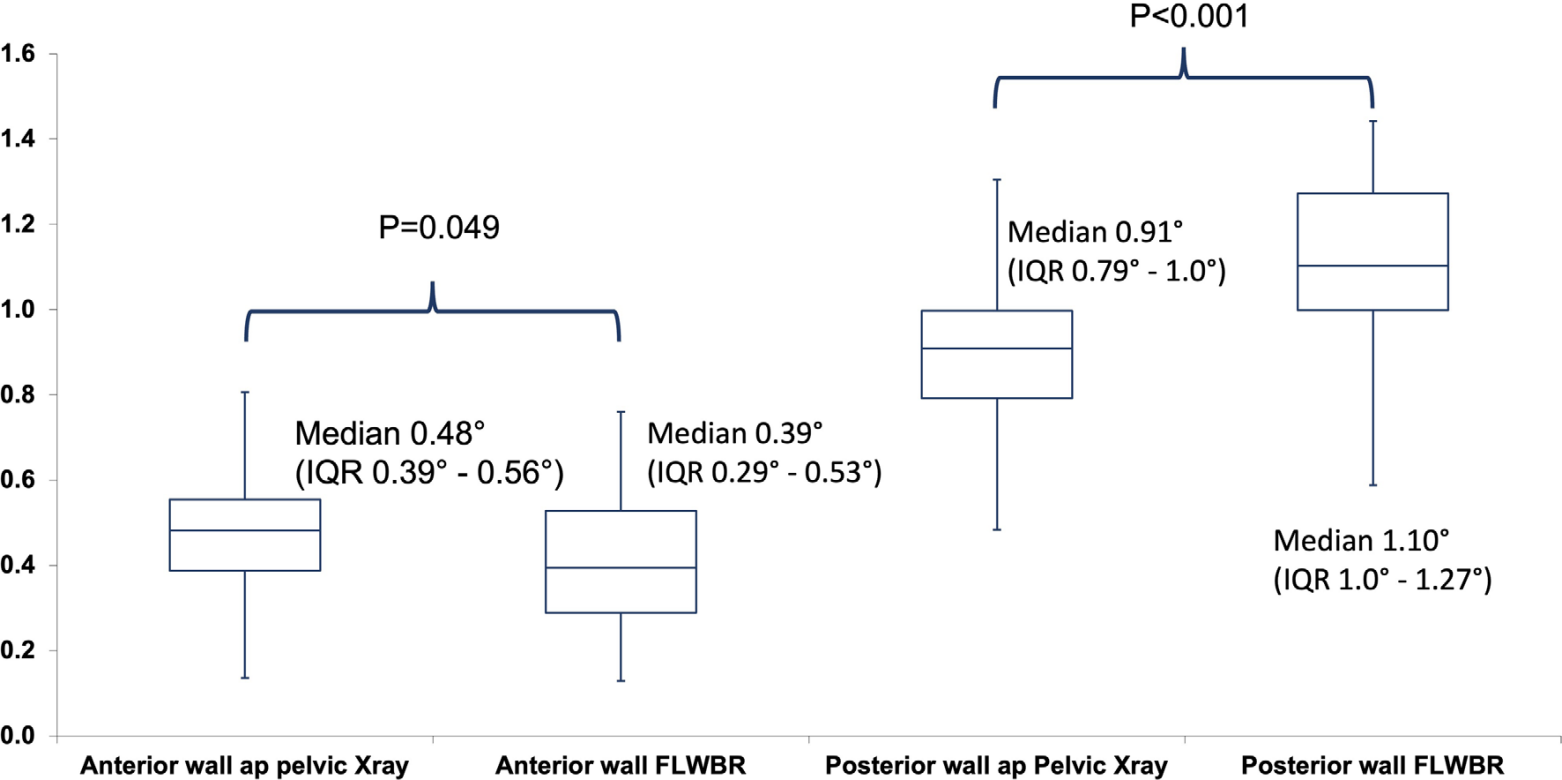
Box plot depicting anterior and posterior wall coverage measured on conventional ap pelvic radiographs and full-length weight-bearing radiographs.

The anterior wall coverage was shown to be significantly reduced in the FLWBR compared to convention ap pelvic X-ray, while posterior coverage was shown to be significantly increased, indicating an anteverted depiction of the acetabulum (Figure 3).

**Figure 4:**
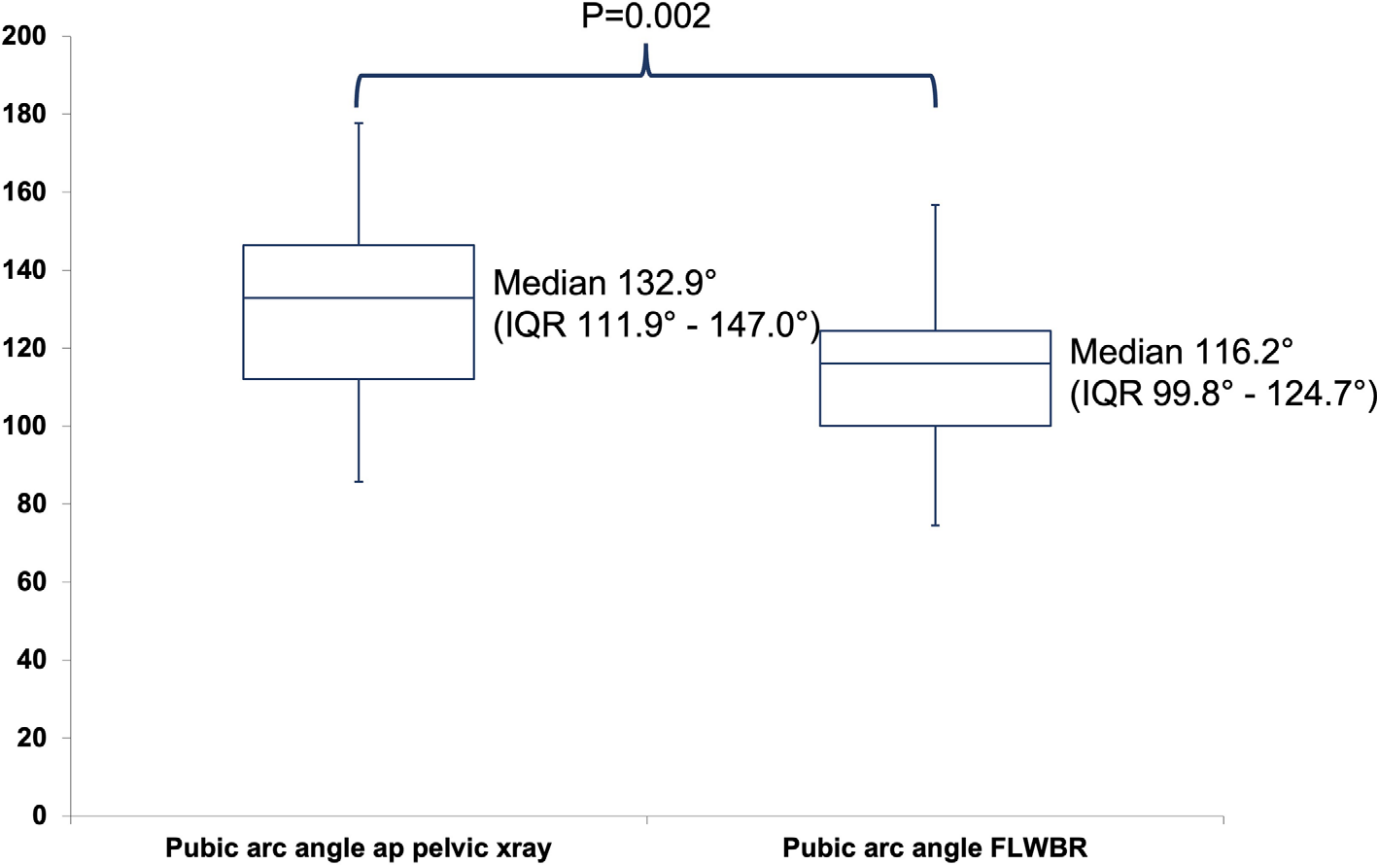
Box plot depicting the pubic-arc angle (subpubic angle) measured on conventional ap pelvic radiographs and full-length weight-bearing radiographs.

The extrusion index did not differ between both radiographic modalities (*p* = 0.31). The pubic arc angle (subpubic angle) was shown to be significantly lower on FLWBR compared to conventional pelvic radiographs (Figure 4).

## Discussion

The most important finding of this study was that FLWBR depicts an anteverted orientation of the acetabulum compared to conventional ap pelvic radiographs. However, the CCD angle of the femur was consistent between both radiographic modalities.

Therefore, the results of this study underline the statement that FLWBR cannot replace conventional ap pelvic radiographs for the evaluation of the morphology of the acetabulum.

This observation may result from several issues associated with a lower centered X-ray beam and the upright standing position of the patient influencing pelvic tilt. A lower-centered X-ray beam was previously recognized as a potential reason for the error in appreciating the anterior acetabular coverage of the femoral head [4, 14]. This would be pronounced in a one-shot FLWBR centered on the knee (as used for this study), and the effect would be more concealed when the FLWBR is conducted in more than one shot and is then fused to an FLWBR image. Furthermore, reduced pelvic tilt in standing position was shown to reduce the anterior coverage resulting in a more dysplastic depiction [15, 16].

Studies with the longest follow-up of periacetabular osteotomy for treating hip dysplasia or impingement surgery have been based on supine ap pelvic radiographs [17-20]. Therefore, it is important to obtain these conventional radiographs in a standardized manner for the overall workup of a painful hip. This is necessary to achieve predictable and comparable results.

This study provides evidence specific to FLWBRs, but it also confirms prior statements regarding variations of acetabular morphology in lower-centered X-ray beams and varying body positions. However, the novelty aspect of this study is underlined by the fact that the CCD angle did not differ between both radiographic modalities. Therefore, FLWBRs are sufficient for evaluating coronal plane malalignment arising from the proximal femur (i.e., coxa valga or vara) – but not evaluating the pelvis or acetabulum.

The clinical relevance of the results is reflected in the planning process of alignment correction of the lower limb. An FLWBR can be safely used to determine coronal plane deformities of the proximal femur.

There are several limitations to this study. Mainly, two factors influenced the depiction of the acetabulum: the position of the X-ray beam and patient posture. It would have been possible to provide a more quantitative measure of the influence of each of the factors by only examining one. This was not possible due to the limited availability of radiographs.

In conclusion, the CCD angle of the femur can be measured on conventional ap radiographs and full-length weight-bearing X-rays for lower limb deformity analysis. However, FLWBR will depict an anteverted acetabular morphology, rendering conventional ap radiographs necessary for planning pelvic osteotomies.

## Conflict of interest

All authors certify that they have no financial conflict of interest with this article.

## Funding

This research did not receive any specific funding.

## Ethical approval

This study received ethical approval from our University’s Ethics committee under protocol 421/2020BO.

## Informed consent

Written informed consent was obtained from all patients.

## Authors contributions

***S.S. Ahmad***: Conceptualization, Methodology, Writing. ***C. Konrads***: Conceptualization, Methodology, Writing. ***A. Steinmeier***: Investigation. ***M Ettinger***: Reviewing, Editing. ***H. Windhagen***: Supervision. ***G.M. Giebel***: Investigation, Writing.
